# Subpopulations Within Juvenile Psoriatic Arthritis: A Review of the Literature

**DOI:** 10.1080/17402520600877802

**Published:** 2006

**Authors:** Matthew L. Stoll, Peter A. Nigrovic

**Affiliations:** 1 Division of Immunology Program in Rheumatology Children's Hospital Boston Boston MA USA; 2 Division of Rheumatology Immunology and Allergy Brigham and Women's Hospital Boston MA USA

## Abstract

The presentation of juvenile psoriatic arthritis (JPsA) has long been recognized to be clinically heterogeneous. As the definition of JPsA expanded to accommodate atypical manifestations of psoriasis in young children, studies began to reflect an increasingly clear biphasic distribution of age of onset, with peaks in the first few years of life and again in early adolescence. These two subpopulations differ in gender ratio, pattern of joint involvement, laboratory findings and potentially response to therapy. Intriguingly, a similar distribution of age of onset has been observed in juvenile rheumatoid arthritis (JRA), and correlates with patterns of HLA association. While a secure classification of subpopulations within JPsA awaits improved pathophysiologic understanding, future research must consider the possibility that different disease mechanisms may be operative in distinct subsets of patients with this disorder.


*Clinical & Developmental Immunology*, June–December 2006; 13(2–4): 377–380

Taylor & Francis

Taylor & Francis Group

### Subpopulations within juvenile psoriatic arthritis: A review of the literature

MATTHEW L STOLL[1] & PETER A NIGROVIC[1,2,†]

[1]*Division of Immunology, Program in Rheumatology, Children's Hospital Boston, Boston, MA, USA, and* [2]*Division of Rheumatology, Immunology and Allergy, Brigham and Women's Hospital, Boston, MA, USA*

**Abstract**
The presentation of juvenile psoriatic arthritis (JPsA) has long been recognized to be clinically heterogeneous. As the definition of JPsA expanded to accommodate atypical manifestations of psoriasis in young children, studies began to reflect an increasingly clear biphasic distribution of age of onset, with peaks in the first few years of life and again in early adolescence. These two subpopulations differ in gender ratio, pattern of joint involvement, laboratory findings and potentially response to therapy. Intriguingly, a similar distribution of age of onset has been observed in juvenile rheumatoid arthritis (JRA), and correlates with patterns of HLA association. While a secure classification of subpopulations within JPsA awaits improved pathophysiologic understanding, future research must consider the possibility that different disease mechanisms may be operative in distinct subsets of patients with this disorder.

**Keywords:** *Juvenile psoriatic arthritis, juvenile rheumatoid arthritis, age of onset, dactylitis*

The diagnosis of juvenile rheumatic conditions has been hampered by the lack of a biological gold standard, thus necessitating phenotypic characterization of different subclasses of arthritis. While existing classification schemes treat JPsA as a single entity, substantial heterogeneity within this group has been noted (Southwood et al. 1989; Petty et al. 2004; Southwood and Petty 2005). Several case series have noted two separate peaks of onset, one within the first few years of life and another in later childhood, raising the possibility that JPsA may actually contain more than one clinical subpopulation (Lambert et al. 1976; Southwood et al. 1989; Stoll et al. 2006). This distinction is important with respect to investigations into the etiology and treatment of this condition. Herein we summarize the data on this issue.

The first documented cases of psoriasis coexisting with arthritis were published by Ansell and Bywaters (1962), in their brief description of 7 children initially diagnosed with JRA who subsequently developed psoriatic skin lesions. The first formal case report of JPsA was published in 1973 by Angevine et al. (1973) detailing a child with psoriasis since the age of three who developed arthritis involving his ankles and small joints of the hands and feet. This report was followed shortly by a case series of 43 children with JPsA, co-authored by Lambert et al. (1976). Observing that arthritis preceded psoriasis in approximately half the cases (Table I), often by several years, these authors expanded the definition of JPsA to include any child with arthritis who developed psoriasis at any time up to 15 years after the onset of arthritis.

Another important finding in the Lambert study was that the age of onset curve appeared to have two peaks. While mean age of onset was 9.3 years, more than 80% of children developed arthritis either before the age of six or after the age of ten. A comparable biphasic age of onset was observed by Truckenbrodt and Hafner (1990) using a similar case definition. Complementing these findings, Ansell et al. (1993)

Correspondence: M. L. Stoll, Division of Immunology, Children's Hospital Boston, 300 Longwood Avenue, Fegan 6, Boston, MA 02115, USA. Tel: 1 617 355 6117. Fax: 1 617 730 0249. E-mail: matthew.stoll@childrens.harvard.edu
[†]E-mail: pnigrovic@partners.org.

ISSN 1740-2522 print/ISSN 1740-2530 online © 2006 Taylor & Francis
DOI: 10.1080/17402520600877802

noted that the distribution of HLA types in children with JPsA differed between those presenting before age 6 and those that presented later in childhood.

Subsequent clinical series of patients with JPsA remained consistent with the observations of Lambert et al. (1976), noting a mean age of onset of 10–12 years of age, generally older than in populations of patients with JRA (Table I) (Calabro 1977; Sills 1980; Shore and Ansell 1982; Hamilton et al. 1990; Truckenbrodt and Hafner 1990). All of these studies confirmed the findings of Lambert et al. that arthritis preceded psoriasis in a substantial proportion of patients (23–58%), often by several years (Table I). These findings prompted several authors to suggest that criteria should be developed to entertain the diagnosis of JPsA in the absence of psoriasis (Sills 1980; Shore and Ansell 1982).

Southwood et al. (1989), answered the call for more inclusive criteria for JPsA, introducing what became known as the Vancouver criteria. These criteria defined *definite JPsA* as arthritis occurring with psoriasis or, in its absence, with at least three of four minor criteria, including dactylitis, nail pitting, family history in a first or second degree relative, or a rash (present by history or on exam) that resembles psoriasis but is not conclusively diagnosed as such. *Probable JPsA* was diagnosed as arthritis without psoriasis, but with exactly two of the minor criteria. In this study, Southwood et al. identified 35 patients with JPsA (24 definite, of whom 21 had psoriasis), reporting a mean age of onset considerably younger than in all of the previous reports (6.7 years). This younger mean age may have reflected an increased ability to detect younger patients, in whom psoriatic skin lesions are often subtle or atypical (Cassidy and Petty 2005). Accordingly, they noted an even more pronounced bimodal distribution, with an early peak (age 0–2) comprised largely of girls, and a later peak (age 12–14) evenly mixed between boys and girls. Notably, using the Lambert criteria, Shore and Ansell (1982) had also noted an earlier age of onset in girls. In support of the notion of JPsA *sine* frank psoriasis, Southwood et al. (1989) reported that the 11 patients with probable JPsA were similar to the 24 with definite JPsA with respect to the patterns of affected joints.

The Vancouver criteria were validated by Roberton et al. (1996), in a study of 63 children with JPsA, of whom 50 had definite JPsA. As with the Southwood study, patients with probable and definite JPsA had similar joints involved at onset and throughout the course of the disease. The population as a whole had a relatively young age of onset of 5.9 years, again consistent with the possibility that including children with more subtle manifestations of the psoriatic diathesis allows for the identification of a population of young patients in addition to the somewhat older children reported in earlier series.

Table I. Case series in JPsA.

| Study | JPsA criteria used | Number of patients (F:M) | Age of onset of arthritis, mean or median (F:M) | Age of onset of psoriasis | % arthritis precedes psoriasis (range of years) |
|---|---|---|---|---|---|
| Ansell and Bywaters, (1962) | Not stated | 7 (3:0)* | 12* | 16.5* | 100% (6–11) |
| Angevine et al. (1973) | Not stated | 1 (0:1) | 12 | 3 | 0% |
| Lambert et al. (1976) | Lambert | 43 (32:11) | 9.3 | 10.4 | 52% (0.25–14) |
| Singsen (1977) | Not stated | 2 (1:1) | 13 (15;11) | 12.5 | 0% |
| Calabro, (1977) | Not stated | 12 (7:5) | Not stated | Not stated | 33% (1–4) |
| Sills (1980) | Lambert | 24 (17:7) | 10.2 | 11.3 | 58% |
| Shore and Ansell (1982) | Lambert | 60 (35:25) | 11–12 (8.2;9.5) | 8.8 | 43%† |
| Wesolowska (1985) | Not stated | 21 (8:13) | 9.7 | Not stated. Present in 18/21 | 48% (mean 3.2 years) |
| Southwood et al. (1989) | Vancouver | 35 (24:11) | 6.7 (bimodal) | Not stated. Present in 21/35 | 48% (1–12.8; mean 5.9) |
| Hamilton et al. (1990) | Lambert | 28 (16:12) | 10.7 | 8.75 | 21% |
| Truckenbrodt and Hafner (1990) | Lambert | 48 (21:27) | 10.7 | 10 | 50% (0.5–12) |
| Roberton et al. (1996) | Vancouver | 63 (44:19) | 5.9 (4.5; 10.1) | Present in 41/63. Range 0.25–20.75 | 56% (mean 3.4 yrs) |
| Stoll et al. (2006) | Vancouver | 139 (82:57) | 7.3 | Not stated. Present in 35/139 | 29% (unpublished data) |

*Limited data available on these 7 patients.; †Range not detailed, although there were 12 cases in which there was a delay between the onset of arthritis and of psoriasis of four or more years.

Using a relatively large cohort of JPsA patients, we set out to study the possibility that younger and older children with JPsA constitute clinically distinct subgroups. We studied 139 children meeting the Vancouver criteria and collected information on joint involvement, laboratory results, and treatment decisions at each visit for each patient (Stoll et al. 2006). We found an age of onset distribution similar to that previously reported, with two peaks: one in early childhood and the other near adolescence, optimally divided at age 5 (Figure 1). The size of our sample enabled us to search for clinical or laboratory differences between older and younger children. Indeed these were quite striking: young children were more likely to be female (76 vs. 50%), to have dactylitis (63 vs. 22%) and polyarticular onset (20 vs. 6%), and to be ANA positive (64 vs. 37%), but were less likely to have frank psoriasis (14 vs. 31%), enthesitis (22 vs. 57%), or axial disease (10 vs. 26%) ($p$ all $<0.05$) (Stoll et al. 2006). Cluster analysis on the 117 (84%) of patients for whom we had complete data also generated two distinct subgroups that differed by age and the above clinical and laboratory variables; interestingly, however, age of onset was a less reliable predictor of clinical phenotype than was the presence of dactylitis, which was present in 100% of the younger cluster ($n = 40$) vs. 1.3% of the older cluster ($n = 77$) ($p < 0.001$) (Stoll et al. 2006). Thus it remains uncertain whether the critical variable determining phenotype in JPsA is truly age or whether age is better regarded as a marker for a particular subset of disease, e.g. arthritis accompanied by dactylitis.

The significance of these dual populations remains uncertain. Very similar general patterns of onset have been observed within JRA, with a peak around

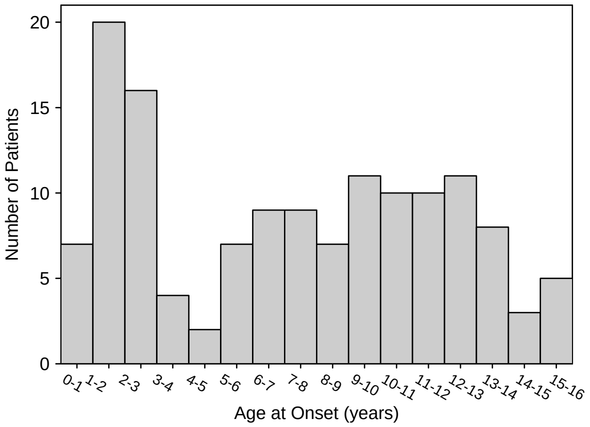

Figure 1. Age of onset of 139 children with JPsA diagnosed under the Vancouver criteria. Evaluation of multiple cutoffs between the younger and older distributions reveals optimal division at $<5$ years vs. 5 years and above; the resulting two distributions do not deviate significantly from normal as assessed by the Lillefors-corrected Kolmogorov-Smirnoff test. Reprinted with permission (pending) from Stoll et al. (2006).

age 2–3 years composed predominantly of girls and a second distribution through mid- and late-childhood where the gender distribution is somewhat more balanced (Sullivan et al. 1975; Murray et al. 1999). Analysis of HLA expression has also reflected a striking variation in allele frequency with age of onset, occasionally in a gender-specific fashion and commonly with a suggestion of an inflection point around age 5–6 years (Murray et al. 1999). It has been hypothesized that early onset arthritis could reflect the influence of endemic infections of childhood (Martini 2003). Indeed, the limited “window of effect” of each HLA allele — the age range during which the allele is associated with an increased or decreased risk of juvenile arthritis — would be consistent with a model in which disease is triggered by antigenic exposures that vary with the age of the child (Murray et al. 1999).

Our finding that age of onset predicts clinical phenotype of JPsA suggests a similar hypothesis of differential susceptibility to environmental exposures, especially given earlier data indicating differences in HLA distribution between younger and older children with JPsA (Ansell et al. 1993; Stoll et al. 2006). It is especially intriguing that the early childhood peak in JRA coincides with that of JPsA, despite clear differences in clinical phenotype, including presence of dactylitis, distribution of joint involvement, and tendency toward a systemic inflammatory state in young patients with JPsA (Huemer et al. 2002; Stoll et al. 2006). This result could suggest an effect of similar age-related exposures such as infections and vaccinations, or potentially a temporally delimited susceptibility within the synovium or enthesis.

An alternate possibility is suggested by the identification by cluster analysis of dactylitis as the best predictor of clinical pattern within our JPsA cohort (Stoll et al. 2006). Dactylitis describes the diffuse swelling of a single digit characteristic of psoriatic arthritis, and may reflect the sum of small joint arthritis, periostitis, tenosynovitis and enthesitis (Olivieri et al. 1996; Kane et al. 1999; Benjamin and McGonagle 2001). While more frequent among younger children, dactylitis was observed among patients of all ages. To the extent that the propensity to develop dactylitis derives from a particular set of pathogenic mechanisms, it could be that the most informative subgrouping of JPsA rests on the presence of this clinical feature rather than the age of onset. If so, then the age of onset curves would likely reflect the net result of unknown biologic and environmental factors promoting forms of psoriatic arthritis with and without dactylitis. Such an approach would have the additional appeal of enabling the search for continuity between pediatric and adult psoriatic arthritis, currently divided by convention at the 16th birthday (Southwood et al. 1989; Petty et al. 2004).

In conclusion, our work has substantiated and extended earlier findings that JPsA is a diverse disease.

We find that these patients may be divided into two subsets according to age of onset, although other clinical features such as the presence of dactylitis might also serve as organizing principles. The significance of these findings for pathogenesis and therapy of JPsA remains uncertain, yet it is clear that future research needs to avoid the assumption that children with JPsA constitute a single homogenous population.

### References

Angevine CD, Pless IB, Baum J, Jacox RF. 1973. Psoriatic arthritis in a child. Arthritis Rheum 16:278–283.

Ansell BM, Bywaters EG. 1962. Diagnosis of “probable” Still’s disease and its outcome. Ann Rheum Dis 21:253–262.

Ansell B, Beeson M, Hall P, Bedford P, Woo P. 1993. HLA and juvenile psoriatic arthritis. Br J Rheumatol 32:836–837.

Benjamin M, McGonagle D. 2001. The anatomical basis for disease localisation in seronegative spondyloarthropathy at entheses and related sites. J Anat 199:503–526.

Calabro JJ. 1977. Psoriatic arthritis in children. Arthritis Rheum 20(S):415–416.

Cassidy JT, Petty RE. 2005. Textbook of pediatric rheumatology. xvi. Philadelphia, PA: Elsevier Saunders. p 792.

Hamilton ML, Gladman DD, Shore A, Laxer RM, Silverman ED. 1990. Juvenile psoriatic arthritis and HLA antigens. Ann Rheum Dis 49:694–697.

Huemer C, Malleson PN, Cabral DA, et al. 2002. Patterns of joint involvement at onset differentiate oligoarticular juvenile psoriatic arthritis from pauciarticular juvenile rheumatoid arthritis. J Rheumatol 29:1531–1535.

Kane D, Greaney T, Bresnihan B, Gibney R, FitzGerald O. 1999. Ultrasonography in the diagnosis and management of psoriatic dactylitis. J Rheumatol 26:1746–1751.

Lambert JR, Ansell BM, Stephenson E, Wright V. 1976. Psoriatic arthritis in childhood. Clin Rheum Dis 2:339–352.

Martini A. 2003. Are the number of joints involved or the presence of psoriasis still useful tools to identify homogeneous disease entities in juvenile idiopathic arthritis? J Rheumatol 30:1900–1903.

Murray KJ, Moroldo MB, Donnelly P, et al. 1999. Age-specific effects of juvenile rheumatoid arthritis-associated HLA alleles. Arthritis Rheum 42:1843–1853.

Olivieri I, Barozzi L, Favaro L, et al. 1996. Dactylitis in patients with seronegative spondylarthropathy. Assessment by ultrasonography and magnetic resonance imaging. Arthritis Rheum 39:1524–1528.

Petty RE, Southwood TR, Manners P, et al. 2004. International League of Associations for Rheumatology classification of juvenile idiopathic arthritis: Second revision, Edmonton, 2001. J Rheumatol 31:390–392.

Roberton DM, Cabral DA, Malleson PN, Petty RE. 1996. Juvenile psoriatic arthritis: Followup and evaluation of diagnostic criteria. J Rheumatol 23:166–170.

Shore A, Ansell BM. 1982. Juvenile psoriatic arthritis—an analysis of 60 cases. J Pediatr 100:529–535.

Sills EM. 1980. Psoriatic arthritis in childhood. Johns Hopkins Med J 146:49–53.

Singsen BH. 1977. Psoriatic arthritis in childhood. Arthritis Rheum 20:408–410.

Southwood TR, Petty RE, Malleson PN, et al. 1989. Psoriatic arthritis in children. Arthritis Rheum 32:1007–1013.

Southwood TR, Petty RE. 2005. Juvenile psoriatic arthritis. In: Cassidy JT, Petty RE, editors. Pediatric rheumatology. Elsevier.

Stoll ML, Zurakowski D, Nigrovic LE, Nichols DP, Sundel RP, Nigrovic PA. 2006. Patients with juvenile psoriatic arthritis comprise two distinct populations. Arthritis Rheum 54:3564–3572.

Sullivan DB, Cassidy JT, Petty RE. 1975. Pathogenic implications of age of onset in juvenile rheumatoid arthritis. Arthritis Rheum 18:251–255.

Truckenbrodt H, Hafner R. 1990. Psoriatic arthritis in childhood. A comparison with subgroups of chronic juvenile arthritis. Z Rheumatol 49:88–94.

Wesolowska H. 1985. Clinical course of psoriatic arthropathy in children. Mater Med Pol 17:185–187.

